# A novel glucose–lipid metabolism–related indicator and its association with metabolic dysfunction–associated fatty liver disease: a cross–sectional study based on a health check–up population

**DOI:** 10.3389/fendo.2026.1770104

**Published:** 2026-04-24

**Authors:** Yunzhen Tang, Zheng Jiang

**Affiliations:** Department of Gastroenterology, The First Affiliated Hospital of Chongqing Medical University, Chongqing, China

**Keywords:** Chinese adults, cholesterol, high density lipoprotein, and glucose index, cross–sectional study, health check-up population, metabolic dysfunction–associated fatty liver disease

## Abstract

**Objective:**

This study aimed to investigate the association between a novel glucose–lipid metabolic indicator, the cholesterol, high density lipoprotein, and glucose (CHG) index, and the prevalence of metabolic dysfunction–associated fatty liver disease (MAFLD), as well as to evaluate its discriminative ability.

**Methods:**

This single–center cross–sectional study included 166,647 adults who underwent a health examination at the Health Management Center of the First Affiliated Hospital of Chongqing Medical University between July 2022 and March 2025. Multivariable logistic regression models were used to assess the association between the CHG index and the prevalence of MAFLD. Restricted cubic spline (RCS) functions were applied to explore potential nonlinear relationships. Receiver operating characteristic (ROC) curve analysis was used to compare the discriminative ability of the CHG index, triglyceride-glucose (TyG) index, and fatty liver index (FLI), and differences in AUCs were assessed using the DeLong test. Subgroup analyses were performed to assess its stability across different populations. Sensitivity analyses were performed to further examine the association between the CHG index and MAFLD.

**Results:**

A total of 166,647 participants were included, comprising 85,643 men (51.4%) and 81,004 women (48.6%), with an overall prevalence of MAFLD of 33.7%. The CHG index was significantly higher in participants with MAFLD than in those without (5.42 ± 0.34 vs. 5.05 ± 0.31; *P* < 0.001). In the fully adjusted model, the CHG index was positively associated with MAFLD (per SD increase in the CHG index: OR = 2.35, 95% CI: 2.31–2.39, *P* < 0.001; Q4 vs Q1: OR = 8.64, 95% CI: 8.21–9.10, *P* < 0.001, *P* for trend < 0.001). Threshold effect analysis demonstrated a nonlinear association between the CHG index and MAFLD (inflection point K = 5.54, *P* for nonlinearity < 0.001). ROC analysis showed that the CHG index had good discriminatory ability for MAFLD (AUC = 0.810, 95% CI: 0.807-0.812), although its performance was lower than that of the TyG index and FLI. Subgroup analyses showed significant interactions between the CHG index and age, sex, and body mass index (BMI) (all *P* for interaction < 0.001), with stronger associations observed among females, younger participants, and those with lower BMI. Sensitivity analyses showed similar results.

**Conclusion:**

The CHG index was positively associated with MAFLD and showed good discriminatory ability in this large Chinese health check-up population. Although its performance was lower than that of the TyG index and FLI, it may still serve as a simple and accessible metabolic indicator for identifying individuals at higher risk of MAFLD in primary healthcare and health screening settings.

## Introduction

Metabolic dysfunction–associated fatty liver disease (MAFLD) was redefined by the international expert consensus in 2020, with its diagnosis established on the presence of hepatic steatosis accompanied by metabolic abnormalities, thereby replacing the exclusion–based diagnostic framework of nonalcoholic fatty liver disease (NAFLD) (1). This updated nomenclature more precisely reflects the metabolic pathogenesis of the disease. The global prevalence of MAFLD is estimated at approximately 38.8%, rendering it the most prevalent chronic liver disease worldwide (2). A proportion of patients with MAFLD may progress to metabolic dysfunction–associated steatohepatitis (MASH), which may subsequently advance to fibrosis, cirrhosis, or hepatocellular carcinoma (HCC) (2). Because of its insidious onset and the absence of effective screening strategies, MAFLD has emerged as a significant global public health burden. Moreover, MAFLD is regarded as the hepatic manifestation of metabolic syndrome and is strongly associated with a heightened risk of extrahepatic comorbidities, including cardiovascular disease, chronic kidney disease, and osteoporosis (3–5).

A major challenge in the management of MAFLD is the achievement of timely and accurate diagnosis. Although liver biopsy remains the gold standard for diagnosing and staging fatty liver disease, it is invasive, costly, and carries risks such as bleeding and infection, thereby limiting its clinical applicability (6). Noninvasive imaging modalities, including ultrasound, FibroScan, and computed tomography (CT), can detect hepatic steatosis or fibrosis; however, their use in large-scale population screening is constrained by issues of accessibility, cost, and feasibility. Therefore, the identification of simple, inexpensive, and reliable biomarkers or composite indices for the noninvasive detection of MAFLD has become an important focus of current research.

To date, several surrogate indices have been proposed for identifying fatty liver or metabolic risk (7–11). Among these, FLI has been widely used as a conventional tool for fatty liver screening, whereas the TyG index has been extensively investigated as a surrogate marker of insulin resistance (IR) and metabolic risk. However, the diagnostic performance and clinical applicability of these indices may vary across different populations and clinical settings, highlighting the need to explore and validate novel indicators with potential utility in MAFLD risk assessment.

The development and progression of MAFLD are closely related to IR and hepatic lipid metabolic dysfunction, which contribute to excessive lipid accumulation in the liver, oxidative stress, and chronic inflammatory responses, and are often accompanied by systemic metabolic disturbances such as gut microbiota dysbiosis (12–14). These alterations not only promote hepatic injury but also overlap with the pathophysiological basis of other metabolic disorders, particularly type 2 diabetes mellitus (T2DM) and cardiovascular disease (CVD) (15, 16). In recent years, the cholesterol, high-density lipoprotein, and glucose (CHG) index has been identified as a novel metabolic indicator which is closely associated with several metabolic and cardiorenal disorders, including T2DM, CVD, diabetic retinopathy, IgA nephropathy, and the cardiorenal–metabolic (CKM) syndrome (17–22). Meanwhile, previous studies have explored the association between the CHG index and the incidence of NAFLD in cohort setting (23). However, the relationship between the CHG index and MAFLD remains unclear, particularly in large-scale health check-up populations, where epidemiological evidence is still limited. Given the evolving transition from NAFLD to MAFLD and the increasing emphasis on metabolic dysfunction in disease definition, further investigation of the potential value of the CHG index in the context of MAFLD is warranted. This study aimed to aimed to evaluate the association between the CHG index and MAFLD prevalence in a large Chinese health examination cohort and to further assess its potential utility as a simple metabolic indicator for identifying individuals at high risk of MAFLD.

## Materials and methods

### Study population

**Figure 1:**
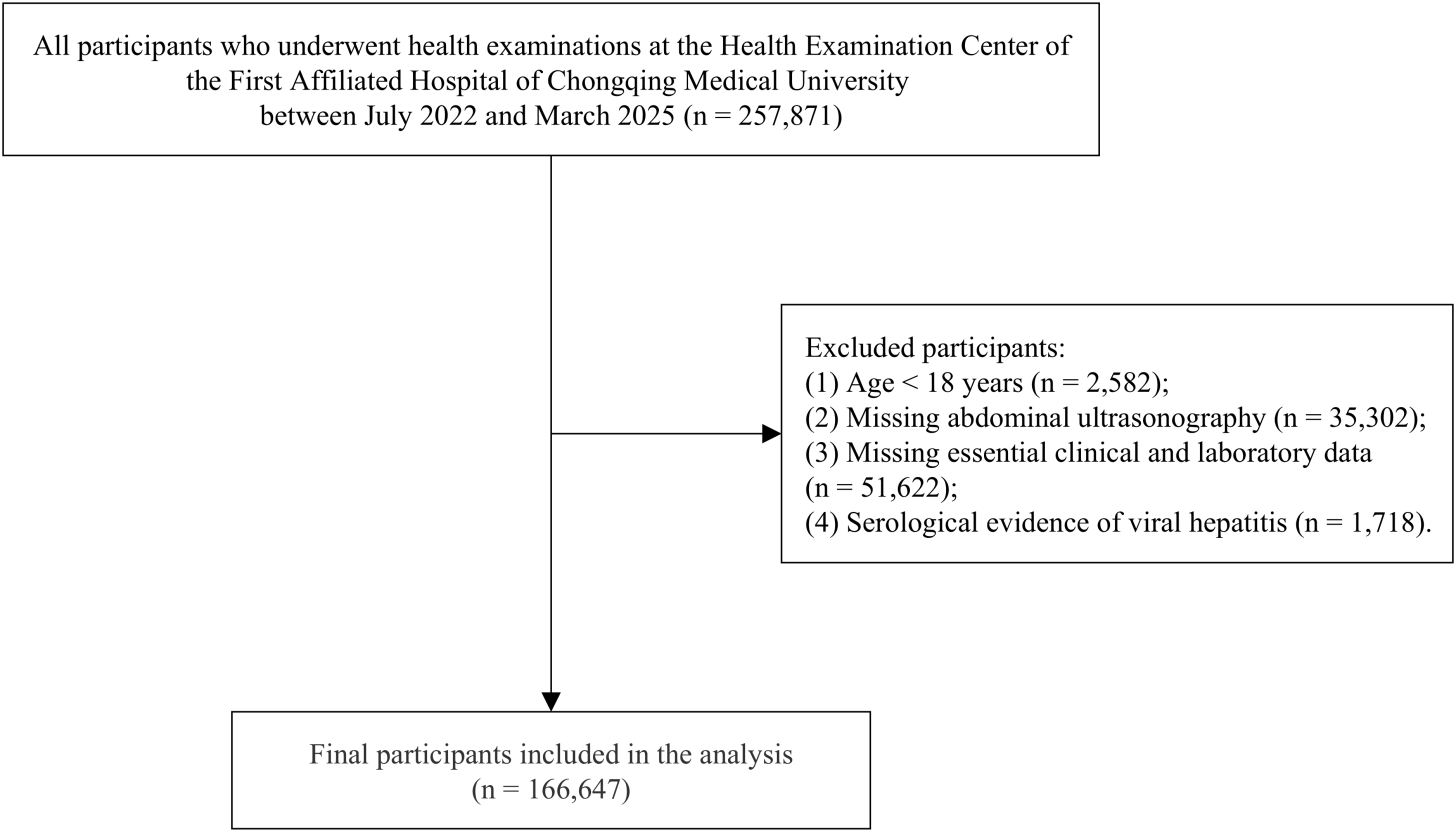
Flowchart of the selection process of study subjects.

This single–center, retrospective cross–sectional study included individuals who underwent health examinations at the Health Management Center of the First Affiliated Hospital of Chongqing Medical University between July 2022 and March 2025. The exclusion criteria were as follows (1): age <18 years (2); missing abdominal ultrasonography (3); missing essential clinical and laboratory data; and (4) serological evidence of viral hepatitis. After applying these criteria, a total of 166,647 eligible participants were included in the final analysis (**Figure 1**). Essential clinical and laboratory data include height, weight, waist circumference, hip circumference, fasting blood glucose, blood lipids, ALT, AST, GGT, and UA.

The study protocol was reviewed and approved by the Institutional Ethics Committee of the First Affiliated Hospital of Chongqing Medical University (approval number: 2025–260–01). Given the retrospective nature of the study, the requirement for informed consent was waived by the committee. All patient data were anonymized prior to analysis, with no identifiable personal information retained. Data confidentiality was strictly maintained, and all study procedures adhered to the principles outlined in the Declaration of Helsinki.

### Data collection

All participants were required to fast for at least 8 hours before undergoing a standardized physical examination. Anthropometric measurements included height (cm), weight (kg), waist circumference (WC, cm), hip circumference (HC, cm), systolic blood pressure (SBP, mmHg), and diastolic blood pressure (DBP, mmHg). Biochemical parameters included alanine aminotransferase (ALT, U/L), aspartate aminotransferase (AST, U/L), γ–glutamyl transpeptidase (GGT, U/L), total cholesterol (TC, mmol/L), triglycerides (TG, mmol/L), high–density lipoprotein cholesterol (HDL–C, mmol/L), low–density lipoprotein cholesterol (LDL–C, mmol/L), uric acid (UA, μmol/L), and fasting plasma glucose (FPG, mmol/L).

All hepatic ultrasonography examinations were performed and interpreted by board–certified radiologists who had received specialized training in hepatobiliary imaging. To minimize diagnostic bias, both the sonographers and interpreting physicians were blinded to the participants’ clinical and laboratory data throughout the imaging acquisition and assessment processes.

### Calculation of metabolic indicators

The CHG index, TyG index, and FLI were calculated according to methods reported in previous literature (11, 20, 24). Unit conversions are as follows: 1 mmol/L = 38.7 mg/dL (TC), 1 mmol/L = 38.7 mg/dL (HDL–C), 1 mmol/L = 88.5 mg/dL (TG) and 1 mmol/L = 18 mg/dL (FPG). The specific formulas are as follows:


$$\text{CHG index}=\text{ln}(\frac{\text{TC}(\text{mg}/\text{dL})\times \text{FPG}(\text{mg}/\text{dL})}{2\times \text{HDL}(\text{mg}/\text{dL})})$$

$$\text{TyG index}=\text{ln}(\frac{\text{TG}(\text{mg}/\text{dL})\times \text{FPG}(\text{mg}/\text{dL})}{2})$$

$$\text{FLI}=\frac{{\text{e}}^{\text{y}}}{1+{\text{e}}^{\text{y}}}\times 100$$

y=0.953×ln(TG(mg/dL))+0.139×BMI+0.718×ln(GGT(U/L))+0.053×WC(cm)−15.745.


$$\text{Waist}-\text{to}-\text{hip ratio }(\text{WHR})=\frac{\text{Waist Circumference}\;(\text{cm})}{\text{Hip Circumference}(\text{cm})}$$

$$\text{Body mass index }(\text{BMI})=\frac{\text{weight}\;(\text{kg})}{\text{height}{\left(\text{m}\right)}^{2}}$$

The diagnosis of MAFLD was established according to the international expert consensus criteria. Participants were required to meet both of the following conditions (1): evidence of hepatic steatosis confirmed by abdominal ultrasonography; and (2) the presence of one or more of the following three conditions: ① BMI ≥23 kg/m² (based on the Asian population standard); ② previously diagnosed T2DM; or ③ at least two metabolic risk abnormalities, defined as follows: waist circumference ≥90 cm for men or ≥80 cm for women; SBP ≥130 mmHg and/or DBP ≥85 mmHg, or current use of antihypertensive medication; plasma TG ≥1.70 mmol/L and/or HDL–C <1.0 mmol/L in men or <1.3 mmol/L in women; FPG level between 5.6 and 6.9 mmol/L or glycated hemoglobin (HbA1c) between 5.7% and 6.4%; high–sensitivity C–reactive protein (hs–CRP) >2 mg/L; homeostatic model assessment of insulin resistance (HOMA–IR) ≥2.5 [1]. Notably, HOMA–IR was not evaluated in the present study. The diagnosis of hepatic steatosis on ultrasonography was based on four established features: (1) hepatorenal echo contrast, (2) increased hepatic echogenicity, (3) posterior beam attenuation, and (4) blurring of vascular margins (25).

### Statistical analysis

All statistical analyses were performed using R software (version 4.4.1; R Foundation for Statistical Computing) and EmpowerStats. All statistical tests were two–sided, and a *p*–value of < 0.05 was considered statistically significant.

Baseline characteristics of participants were stratified according to MAFLD status. Continuous variables are presented as mean ± standard deviation or median (Q1–Q3), while categorical variables are presented as frequency (percentage). The normality of continuous variables was assessed using histograms and the Shapiro–Wilk test. Differences between the two groups for normally distributed variables were compared using independent samples t-test, while the Wilcoxon rank-sum test was used for non–normally distributed variables. The Chi–square test was used for comparing categorical variables.

Multicollinearity was assessed, and the variance inflation factors (VIFs) for all covariates were below 5, indicating no significant multicollinearity. Univariable and multivariable logistic regression analyses were employed to examine the association between the CHG index and the prevalence of MAFLD. Model I was unadjusted, Model II was adjusted for age and sex, Model III was adjusted for age, sex, HC, SBP, DBP, ALT, AST, GGT and UA. To evaluate the dose–response relationship between the CHG index and MAFLD, a restricted cubic spline (RCS) model was fitted to explore potential non–linearity. Furthermore, a two–piecewise linear regression model was used to examine the threshold effect and identify the inflection point (K–value). Receiver operating characteristic (ROC) curve analysis was utilized to compare the discriminative ability of the CHG index, TyG index, and FLI for MAFLD. The AUC was calculated for each index, and differences between AUCs were compared using the DeLong test. Bootstrap resampling with 1,000 iterations was used for internal validation, and calibration curves were generated to assess the agreement between predicted and observed outcomes. Subgroup analyses were further conducted by stratifying participants based on sex, age, and BMI. Sensitivity analyses were conducted to assess the robustness of the main findings. First, additional adjustments were made for TG and LDL-C. Second, alternative definitions of MAFLD were applied by excluding the BMI criterion or glucose-related diagnostic components, and multivariable logistic regression analyses were repeated accordingly.

## Results

### Baseline characteristics of study participants

A total of 166,647 participants were included in this study, comprising 85,643 men (51.4%) and 81,004 women (48.6%), with a median age of 43 years. Participants were classified into two groups according to the presence or absence of MAFLD. Among them, 56,256 participants (33.7%) were diagnosed with MAFLD. The baseline characteristics of the two groups are presented in **Table 1**.

**Table 1:** Baseline characteristics of the study population.

Variables	Total n = 166,647	Non–MAFLD n = 110,391	MAFLD n = 56,256	*P* value
Age (years)	43.00 (34.00, 55.00)	41.00 (33.00, 53.00)	47.00 (37.00, 57.00)	<0.001
Gender, n (%)				<0.001
Male	85,643 (51.39)	44,681 (40.48)	40,962 (72.81)	
Female	81,004 (48.61)	65,710 (59.52)	15,294 (27.19)	
CHG index	5.17 ± 0.37	5.05 ± 0.31	5.42 ± 0.34	<0.001
TyG index	8.67 ± 0.67	8.42 ± 0.54	9.15 ± 0.64	<0.001
FLI	20.39 (7.00, 46.66)	10.45 (4.55, 23.16)	53.21 (34.37, 72.68)	<0.001
BMI (kg/m2)	23.86 ± 3.35	22.50 ± 2.72	26.53 ± 2.80	<0.001
Weight (kg)	64.07 ± 12.21	59.50 ± 9.97	73.05 ± 11.17	<0.001
Height (cm)	163.44 ± 8.51	162.31 ± 8.25	165.65 ± 8.57	<0.001
WHR	0.85 ± 0.08	0.83 ± 0.07	0.91 ± 0.06	<0.001
Waist circumference (cm)	80.61 ± 10.35	76.39 ± 8.79	88.89 ± 7.86	<0.001
Hip circumference (cm)	94.12 ± 6.13	92.28 ± 5.43	97.75 ± 5.80	<0.001
AST (U/L)	19.00 (16.00, 24.00)	18.00 (16.00, 22.00)	22.00 (18.00, 27.00)	<0.001
ALT (U/L)	18.00 (13.00, 28.00)	15.00 (11.00, 22.00)	27.00 (19.00, 40.00)	<0.001
GGT (U/L)	22.00 (15.00, 25.00)	17.00 (13.00, 24.00)	25.00 (18.00, 37.00)	<0.001
UA (μmol/L)	348.01 ± 94.17	321.97 ± 83.14	399.10 ± 93.61	<0.001
HDL–C (mmol/L)	1.39 ± 0.34	1.49 ± 0.34	1.20 ± 0.26	<0.001
LDL–C(mmol/L)	2.86 ± 0.80	2.80 ± 0.77	2.99 ± 0.84	<0.001
Total Cholesterol (mmol/L)	5.01 ± 1.00	4.91 ± 0.96	5.20 ± 1.05	<0.001
Triglycerides (mmol/L)	1.28 (0.88, 1.93)	1.05 (0.77, 1.48)	1.95 (1.40, 2.80)	<0.001
Fasting plasma glucose (mmol/L)	5.20 (4.90, 5.60)	5.10 (4.90, 5.50)	5.50 (5.10, 6.10)	<0.001
Systolic blood pressure (mmHg)	122.24 ± 18.56	118.03 ± 17.63	130.50 ± 17.54	<0.001
Diastolic blood pressure (mmHg)	75.40 ± 10.80	72.79 ± 9.94	80.53 ± 10.58	<0.001

Continuous variables conforming to a normal distribution are presented as mean ± standard deviation (Mean ± SD), otherwise, they are expressed as median and quantile (Median (IQR)), and categorical variables are presented as percentages.

MAFLD, metabolic associated fatty liver disease; CHG, cholesterol, high–density lipoprotein, and glucose; TyG, triglyceride-glucose index; FLI, fatty liver index; BMI, body mass index; WHR, waist–to–hip ratio; ALT, alanine aminotransferase; AST, aspartate aminotransferase; GGT, gamma glutamyl transferase; UA, uric acid; HDL–C, high–density lipoprotein cholesterol; LDL–C, low–density lipoprotein cholesterol.

Compared with participants without MAFLD, those in the MAFLD group were older, had a higher proportion of men, and exhibited significantly higher values of BMI, WC, WHR, SBP, and DBP (all *P* < 0.001).

With respect to laboratory parameters, participants with MAFLD had higher levels of ALT, AST, GGT, UA, LDL–C, TC, TG, and FPG, as well as lower levels of HDL–C (all *P* < 0.001). The CHG index, TyG index and FLI of the MAFLD group were significantly higher than those of the non-MAFLD group (all *P* < 0.001).

### Association between CHG index and MAFLD

Univariate and multivariate logistic regression analyses demonstrated a positive association between the CHG index and the risk of MAFLD (**Table 2**). In the unadjusted model, each one–standard deviation (SD) increase in the CHG index was associated with approximately a 3.94–fold higher risk of MAFLD (OR = 3.94, 95% CI: 3.85–4.03, *P* < 0.001). This positive association remained significant in the fully adjusted model (OR = 2.35, 95% CI: 2.31–2.39, *P* < 0.001). VIF analysis confirmed the absence of severe multicollinearity among the CHG index and all adjusted covariates (**Supplementary Table 1**).

**Table 2:** Association of CHG index with MAFLD.

Exposure	OR (95%CI), *P* value		
	Model I	Model II	Model III
CHG (per SD increase)	3.94 (3.85 – 4.03) <0.001	3.43 (3.38 – 3.49) <0.001	2.35 (2.31 – 2.39) <0.001
CHG (quartile)			
Q1	1.0	1.0	1.0
Q2	3.82 (3.64 – 4.00)	3.19 (3.04 – 3.34)	2.19 (2.08 – 2.30)
Q3	11.11 (10.62 – 11.62)	8.15 (7.79 – 8.54)	4.16 (3.96 – 4.37)
Q4	33.44 (31.96 – 34.99)	22.30 (21.28 – 23.37)	8.64 (8.21 – 9.10)
*P* for trend	<0.001	<0.001	<0.001

Model I: no covariate adjustment.

Model II: adjusted for sex, age.

Model III: adjusted for sex, age, HC, SBP, DBP, ALT, AST, GGT, UA.

OR, odds ratio; CI, confidence interval; MAFLD, metabolic associated fatty liver disease; CHG, cholesterol, high–density lipoprotein, and glucose; HC, hip circumference; SBP, systolic blood pressure; DBP, diastolic blood pressure; ALT, alanine aminotransferase; AST, aspartate aminotransferase; GGT, gamma glutamyl transferase; UA, uric acid.

When the CHG index was categorized into quartiles, the risk of MAFLD increased progressively across quartile groups (*P* for trend < 0.001). In the fully adjusted model, participants in the fourth quartile (Q4) had an approximately 8.64-fold higher risk of MAFLD compared to participants in the lowest quartile (Q1) (Q4 vs Q1: OR = 8.64, 95% CI: 8.21–9.10) (**Table 2**).

### Dose–response relationship between the CHG index and MAFLD

**Figure 2:**
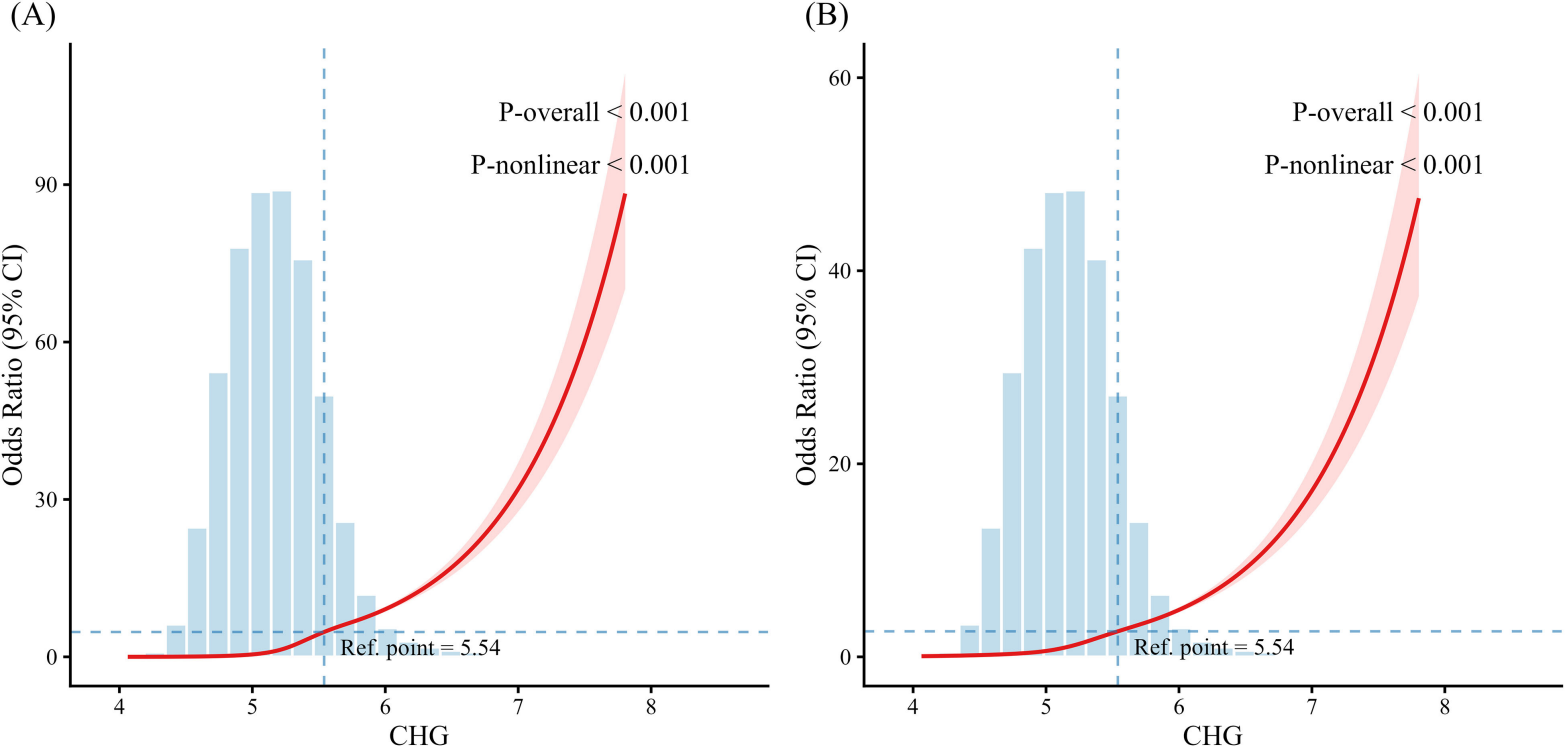
Restricted cubic spline analysis of the association between the CHG index and MAFLD **(A)** Unadjusted models; **(B)** Models adjusted for sex, age, HC, SBP, DBP, ALT, AST, GGT, UA. MAFLD, metabolic associated fatty liver disease; CHG, cholesterol, high–density lipoprotein, and glucose; CI, confidence interval; HC, hip circumference; SBP, systolic blood pressure; DBP, diastolic blood pressure; ALT, alanine aminotransferase; AST, aspartate aminotransferase; GGT, gamma glutamyl transferase; UA, uric acid.

RCS analysis was performed to examine the dose–response relationship between the CHG index and MAFLD prevalence (**Figure 2**). Five knots were placed at the 5th, 27.5th, 50th, 72.5th, and 95th percentiles of the CHG index to assess the potential non-linear association. **Figure 2A** shows a significant non-linear association between the CHG index and the risk of MAFLD in the unadjusted model (*P* for overall < 0.001; *P* for nonlinearity < 0.001). After adjusting for age, sex, HC, SBP, DBP, ALT, AST, GGT and UA (**Figure 2B**), this non-linear trend remained statistically significant (*P* for overall < 0.001; *P* for nonlinearity < 0.001).

### Threshold effect of the CHG index on MAFLD prevalence

To further assess the potential nonlinear relationship between the CHG index and MAFLD prevalence, a two–piecewise logistic regression model was employed (**Table 3**). In this model, the ORs indicate the risk of MAFLD for each 1-unit increase in the CHG index. The results revealed a clear threshold effect between the two variables (log–likelihood ratio test, *P* < 0.001). The model identified an inflection point (K value) at 5.54 which has been marked in **Figure 2**. When the CHG index was below this threshold, each one-unit increase was associated with a higher risk of MAFLD (OR = 16.52, 95% CI: 15.50–17.61, *P* < 0.001). Beyond the threshold of 5.54, the association remained significant but was attenuated (OR = 3.03, 95% CI: 2.71–3.39, *P* < 0.001).

**Table 3:** Threshold effect analysis of CHG index on MAFLD prevalence.

Metabolic dysfunction–associated fatty liver disease	Adjusted OR (95% CI) *P* value
Fitting by the standard logistic regression	10.42 (9.93, 10.93) <0.001
Fitting by the two–piecewise logistic regression	
Inflection point (K)	5.54
CHG < K effect	16.52 (15.50, 17.61) <0.001
CHG > K effect	3.03 (2.71, 3.39) <0.001
Log likelihood ratio	<0.001

The ORs and 95% CIs represent the risk of MAFLD per 1-unit increase in the CHG index. The model was fully adjusted for sex, age, HC, SBP, DBP, ALT, AST, GGT, and UA.

MAFLD, metabolic associated fatty liver disease; CHG, cholesterol, high–density lipoprotein, glucose; OR, odds ratio; CI, confidence interval; HC, hip circumference; SBP, systolic blood pressure; DBP, diastolic blood pressure; ALT, alanine aminotransferase; AST, aspartate aminotransferase; GGT, gamma glutamyl transferase; UA, uric acid.

### Performance of the CHG index, TyG index and FLI

**Figure 3:**
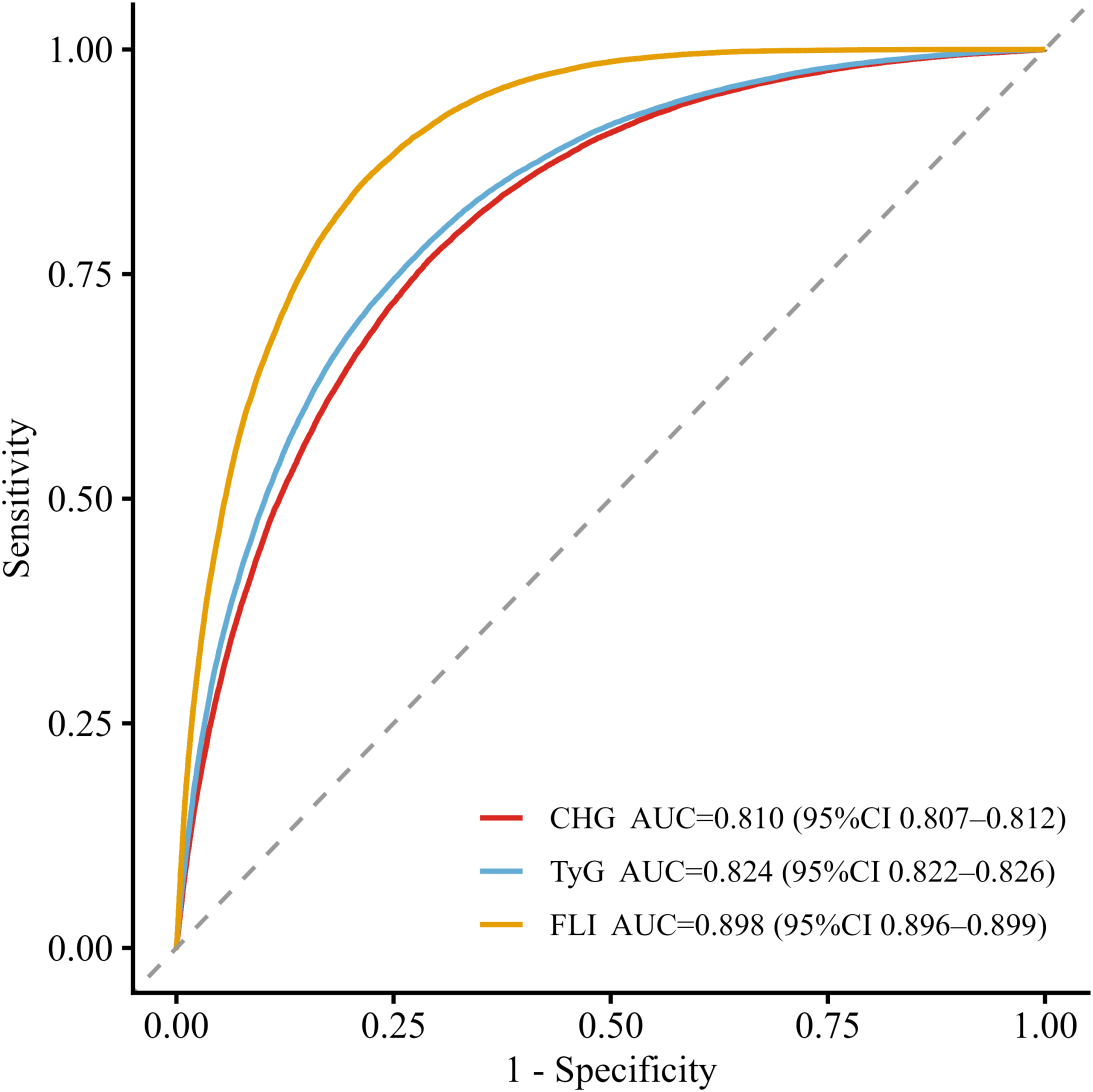
ROC curves of the CHG index, TyG index and FLI for MAFLD ROC curve of the CHG index, TyG index and FLI for MAFLD risk assessment. The x-axis represents 1–specificity, and the y-axis represents sensitivity. ROC, receiver operating characteristic; CI, confidence interval; MAFLD, metabolic associated fatty liver disease; CHG, cholesterol, high–density lipoprotein, and glucose; TyG, triglyceride-glucose index; FLI, fatty liver index.

**Figure 4:**
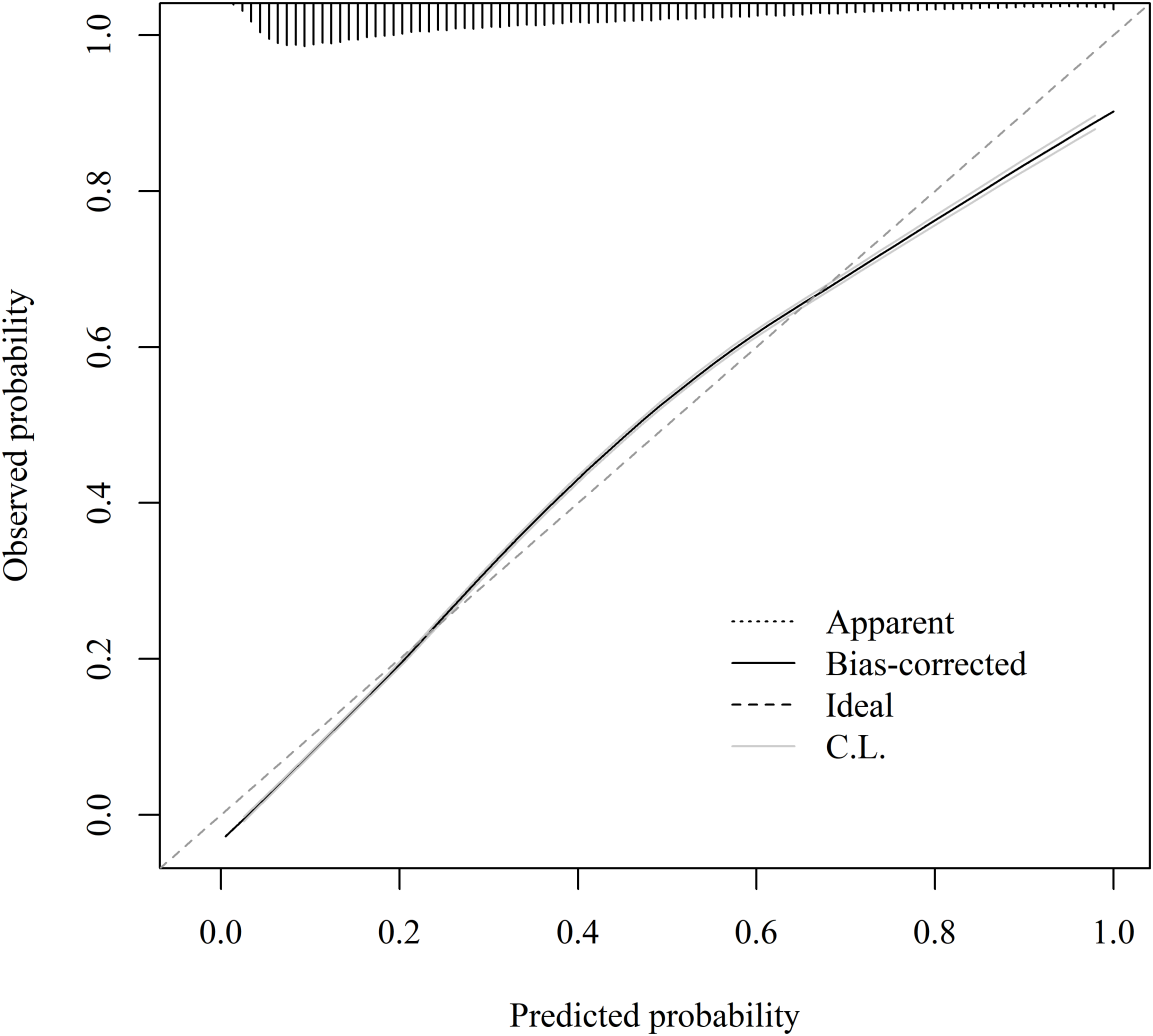
Calibration curve of the CHG index for MAFLD CHG, cholesterol, high-density lipoprotein, and glucose; MAFLD, metabolic dysfunction-associated fatty liver disease; C.L, confidence limits.

ROC curve analysis was performed to compare the ability of CHG, TyG, and FLI to discriminate MAFLD (**Figure 3**). The results showed that all three indices had discriminative value for MAFLD, with FLI demonstrating the best overall performance. The AUC of FLI was 0.898 (95% CI: 0.896–0.899), which was higher than that of CHG index (AUC = 0.810, 95% CI: 0.807–0.812) and TyG index (AUC = 0.824, 95% CI: 0.822–0.826). **Table 4** presents the detailed discriminative performance metrics for each index. Pairwise comparisons based on ΔAUC, NRI, and IDI are provided in **Supplementary Table 2**. The calibration curve of the CHG model was generated using 1,000 bootstrap resamples and demonstrated good agreement between the estimated and observed probabilities (**Figure 4**).

**Table 4:** Discriminative performance of the CHG index, TyG index and FLI for MAFLD.

Index	AUC (95% CI)	Cut-off value	Sensitivity	Specificity	Youden index	PPV	NPV
CHG	0.810 (0.807–0.812)	5.197	0.765	0.709	0.475	0.573	0.856
TyG	0.824 (0.822–0.826)	8.709	0.763	0.732	0.495	0.592	0.858
FLI	0.898 (0.896–0.899)	25.714	0.856	0.782	0.637	0.666	0.914

CHG, cholesterol, high-density lipoprotein, and glucose; TyG, triglyceride-glucose; FLI, fatty liver index; MAFLD metabolic associated fatty liver disease; AUC, area under the curve; CI, confidence interval; PPV, positive predictive value; NPV, negative predictive value.

### Subgroup analysis

**Figure 5:**
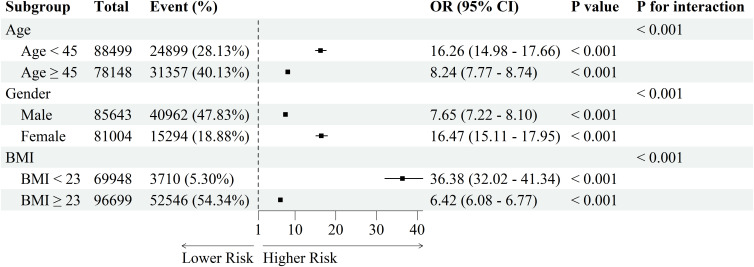
Subgroup analysis of the association between CHG index and MAFLD ORs and 95% CIs were calculated per 1-unit increase in the CHG index. CHG, cholesterol, high-density lipoprotein, and glucose; MAFLD, metabolic dysfunction-associated fatty liver disease; BMI, body mass index; OR, odds ratio; CI, confidence interval.

To explore the stability and interaction effects of the association between the CHG index and MAFLD across different populations, stratified subgroup analyses were conducted according to age, sex, and BMI (**Figure 5**). Significant interactions were observed across all subgroups (all *P* for interaction < 0.001). All ORs represent the effect per 1-unit increase in the CHG index. The association between the CHG index and MAFLD was stronger among women, people under 45 years of age, and non-obese populations.

### Sensitivity analysis

Sensitivity analyses were conducted to evaluate the robustness of the association between the CHG index and MAFLD. First, additional adjustments for lipid-related variables, including TG and LDL-C, did not materially alter the observed association (**Supplementary Table 3**).

Second, to minimize potential circularity related to the diagnostic criteria of MAFLD, we conducted additional sensitivity analyses by modifying the definition of MAFLD. After excluding the BMI criterion and glucose-related criteria, the univariate and multivariate logistic regression analyses were repeated, and the results were similar to those of the primary analyses (**Supplementary Tables 4**, **5**).

## Discussion

Based on data from a large health check-up population, this study evaluated the association between the CHG index and MAFLD. Our findings indicate that the CHG index is independently associated with an increased risk of MAFLD and exhibits a nonlinear dose–response relationship. The CHG index and MAFLD risk exhibit a threshold effect; beyond the threshold, the risk continues to increase, but the rate of increase is markedly attenuated.

In the ROC analysis, the CHG index showed good discriminative ability for identifying MAFLD. However, its performance was lower than that of the TyG index and FLI. The FLI requires a relatively complex calculation and incorporates liver enzyme measurements, which may limit its applicability in primary healthcare settings. Although the discriminative ability of the CHG index was slightly lower than that of the TyG index, it may still provide useful information for identifying individuals with MAFLD. Since this study was conducted in a Chinese health check-up population, the generalizability of these findings to other ethnic groups requires further validation.

Notably, subgroup analyses showed that the association between the CHG index and MAFLD varied across different populations. The association was stronger in women than in men, which may be related to sex hormone–mediated differences in glucose metabolism, fat distribution, and insulin sensitivity in adipose and hepatic tissues. Under physiological conditions, estrogen exerts protective effects by limiting fat accumulation and suppressing inflammation. However, when estrogen levels decline, this protective effect is weakened, resulting in greater visceral fat deposition and amplified metabolic disturbances associated with elevated CHG levels (26, 27). The association was also more pronounced among individuals aged <45 years, suggesting a potential age-related difference in the association between the CHG index and MAFLD. One possible explanation is that, in older individuals, the association between the CHG index and MAFLD may be attenuated by the metabolic background associated with aging. Furthermore, a stronger association was also observed among participants with BMI < 23 kg/m². However, this finding should be interpreted cautiously. In our study, the prevalence of MAFLD in this subgroup only 5.3%, which may have led to sparse data and reduced model stability, thereby contributing to the unusually large odds ratio and wide confidence interval. Nevertheless, this finding may still suggest that metabolic abnormalities captured by the CHG index are relevant in individuals without overt obesity, a population in whom MAFLD risk may be underestimated in routine screening. Previous studies have shown that lean MAFLD is not only associated with visceral adiposity but also with sarcopenia and myosteatosis (28, 29). These individuals often exhibit impaired fatty acid oxidation and skeletal muscle insulin resistance, which promote hepatic lipid deposition. In addition, genetic susceptibility, such as genes like PNPLA3 and TM6SF2, has been reported to be associated with the development of lean MAFLD (30).

The sensitivity analyses further supported the robustness of the association between the CHG index and MAFLD. After additional adjustment for TG and LDL-C, the association remained statistically significant, suggesting that the observed association was not simply due to these closely related lipid parameters. Second, when modified MAFLD definitions excluding the BMI criterion or glucose-related diagnostic criteria were applied, the association between the CHG index and MAFLD also remained significant. These findings indicate that the observed association was not merely a reflection by overlap between the components of the CHG index and the diagnostic criteria for MAFLD.

Although the biological basis linking the CHG index to MAFLD has not been fully clarified, this association may be related to the three metabolic components included in the index: TC, HDL-C, and FPG. These indicators reflect the body’s lipid and glucose metabolism and summarize overall metabolic status. An elevated CHG index reflects a state of metabolic imbalance characterized by increased cholesterol levels, reduced HDL–C, and abnormal glucose regulation, which is closely linked to IR and metabolic dysfunction. This pathophysiological process is closely related to the mechanisms of MAFLD: IR promotes excessive hepatic lipid uptake and synthesis, leading to intrahepatic accumulation of triglycerides and cholesterol (31). When hepatocytes fail to efficiently metabolize lipids, lipotoxicity may occur, accompanied by endoplasmic reticulum stress, mitochondrial dysfunction, and reactive oxygen species production, thereby aggravating hepatocellular injury (32). Persistent hyperglycemia may further enhance oxidative stress and inflammatory responses. Hyperglycemia and dyslipidemia, both downstream consequences of IR, activate inflammatory signaling pathways, promoting the release of proinflammatory cytokines from hepatic and adipose tissues, thereby sustaining chronic low–grade inflammation and aggravating liver injury, ultimately contributing to the progression of MASH (33).

Overall, IR appears to play a central role of MAFLD development, amplifying hepatic lipid accumulation, oxidative stress, chronic inflammation, and mitochondrial dysfunction through synergistic effects of dyslipidemia and hyperglycemia, forming a vicious cycle (34). The CHG index may reflect the effects of these interrelated metabolic disturbances, and previous studies have reported that it demonstrates better discriminative ability than traditional indices for metabolic disorders (20). Our study found that participants with higher CHG index values had an increased risk of MAFLD. These findings suggest that the CHG index, as a comprehensive metabolic biomarker, reflect the degree of IR and the risk of MAFLD to some extent.

Nevertheless, several limitations should be acknowledged. Firstly, as a cross–sectional study, causal inferences cannot be established. Second, some degree of conceptual overlap exists between the components of the CHG index and the diagnostic criteria for MAFLD, which may introduce potential incorporation bias. Third, although multiple covariates were adjusted for, residual confounding cannot be fully excluded, such as smoking, alcohol intake, physical activity, medication use, HbA1c, insulin, and other potential confounders. Fourth, since the study population was derived from a health examination cohort, potential selection bias cannot be excluded. Third, MAFLD diagnosis was based on ultrasonography, which precludes differentiation of disease stages or inflammatory severity. Future studies incorporating longitudinal follow–up and quantitative imaging assessments are warranted to validate the predictive value and underlying mechanisms of the CHG index in the context of MAFLD.

## Conclusion

This study demonstrated a positive association between the CHG index and MAFLD in a Chinese adult population, with acceptable discriminative ability. Although its performance was lower than that of the TyG index and FLI, the CHG index may still serve as a simple metabolic indicator for identifying individuals with a greater probability of MAFLD. Furthermore, incorporating the CHG index into multivariable risk assessment models for high-risk populations may improve the accuracy of MAFLD screening in primary healthcare settings and health checkups.

## Data Availability

The raw data supporting the conclusions of this article will be made available by the authors, without undue reservation.

## Glossary

CHG: cholesterol, high–density lipoprotein, and glucose
TyG: triglyceride-glucose index
FLI: fatty liver index
MAFLD: metabolic dysfunction–associated fatty liver disease
MASH: metabolic dysfunction–associated steatohepatitis
T2DM: type 2 diabetes mellitus
CVD: cardiovascular disease
TC: total cholesterol
TG: triglyceride
LDL–C: low–density lipoprotein cholesterol
HDL–C: high–density lipoprotein cholesterol
WC: waist circumference
HC: hip circumference
BMI: body mass index
SBP: systolic blood pressure
DBP: diastolic blood pressure
AST: aspartate aminotransferase
ALT: alanine aminotransferase
GGT: gamma–glutamyl transferase
UA: uric acid
FPG: fasting plasma glucose
OR: odds ratio
CI: confidence interval
ROC: receiver operating characteristic
RCS: estricted cubic spline
AUC: area under the curve.
