# Supplementary material for: A novel glucose–lipid metabolism–related indicator and its association with metabolic dysfunction–associated fatty liver disease: a cross–sectional study based on a health check–up population
Source: Front Endocrinol (Lausanne). 2026 Apr 24;17:1770104. doi: 10.3389/fendo.2026.1770104 (PMC13152771; doi:10.3389/fendo.2026.1770104)
Supplement: Supplementary file 1 [file Table1.docx]

**A Novel Glucose–Lipid Metabolism–related Indicator and Its Association with Metabolic Dysfunction–associated Fatty Liver Disease: A Cross–sectional Study Based on a health check–up population**

**Supplementary Materials**

[**Table S1** Variance inflation factors for multicollinearity assessment 2](#_Toc13092)

[**Table S2** Comparison of the discriminative performance of CHG, TyG, and FLI for identifying MAFLD 3](#_Toc29426)

[**Table S3** Sensitivity analysis with additional adjustment for TG and LDL-C 4](#_Toc17702)

[**Table S4** Association between the CHG index with MAFLD after excluding the BMI criterion 5](#_Toc13217)

[**Table S5** Association between the CHG index with MAFLD after excluding glucose–related criteria 6](#_Toc11035)

**Table S1** Variance inflation factors for multicollinearity assessment

| **Variable** | **VIF** |
| --- | --- |
| CHG | 1.49 |
| Sex | 1.63 |
| Age | 1.44 |
| HC | 1.33 |
| SBP | 2.71 |
| DBP | 2.35 |
| ALT | 3.41 |
| AST | 3.02 |
| GGT | 1.38 |
| UA | 1.70 |

Note: VIF, variance inflation factor; CHG, cholesterol, high–density lipoprotein, and glucose; HC, hip circumference; SBP, systolic blood pressure; DBP, diastolic blood pressure; ALT, alanine aminotransferase; AST, aspartate aminotransferase; GGT, gamma-glutamyl transferase; UA, uric acid.

**Table S2** Comparison of the discriminative performance of CHG, TyG, and FLI for identifying MAFLD

| **Comparison** | **ΔAUC (95% CI)** | ***P* value** | **NRI (95% CI)** | ***P* value** | **IDI (95% CI)** | ***P* value** |
| --- | --- | --- | --- | --- | --- | --- |
| TyG vs. CHG | 0.014 (0.012-0.016) | <0.001 | 0.155 (0.145-0.165) | <0.001 | 0.028 (0.026-0.030) | <0.001 |
| FLI vs. CHG | 0.088 (0.086-0.090) | <0.001 | 0.742 (0.732-0.753) | <0.001 | 0.181 (0.178-0.183) | <0.001 |

Note: ΔAUC, NRI, and IDI were calculated as the difference between the first and second indices in each comparison.

Abbreviations: CHG, cholesterol, high-density lipoprotein, and glucose; TyG, triglyceride-glucose; FLI, fatty liver index; MAFLD, metabolic associated fatty liver disease; AUC, area under the receiver operating characteristic curve; NRI, net reclassification index; IDI, integrated discrimination improvement。

**Table S3** Sensitivity analysis with additional adjustment for TG and LDL-C

| **Exposure** | **OR (95% CI), *P* value** | | | |
| --- | --- | --- | --- | --- |
|  | **Model I** | **Model II** | **Model III** | **Model IV** |
| CHG (per SD increase) | 2.35 (2.31–2.39) <.001 | 1.95 (1.92–1.99) <.001 | 2.76 (2.70–2.81) <.001 | 2.30 (2.25–2.36) <.001 |
| CHG (quartiles) |  |  |  |  |
| Q1 | 1.0 |  | 1.0 | 1.0 |
| Q2 | 2.19 (2.08–2.30) | 2.01 (1.91–2.12) | 2.72 (2.58–2.87) | 2.36 (2.23–2.49) |
| Q3 | 4.16 (3.96–4.37) | 3.41 (3.24–3.59) | 6.00 (5.69–6.33) | 4.50 (4.26–4.76) |
| Q4 | 8.64 (8.21–9.10) | 5.50 (5.21–5.81) | 13.72 (12.96–14.52) | 8.14 (7.64–8.67) |
| *P* for trend | <.001 | <.001 | <.001 | <.001 |

Model I: adjusted for sex, age, HC, SBP, DBP, ALT, AST, GGT, UA；

Model II: adjusted for sex, age, HC, SBP, DBP, ALT, AST, GGT, UA, TG；

Model III: adjusted for sex, age, HC, SBP, DBP, ALT, AST, GGT, UA, LDL–C；

Model IV: adjusted for sex, age, HC, SBP, DBP, ALT, AST, GGT, UA, TG, LDL–C；

Abbreviations: OR, odds ratio; CI, confidence interval; MAFLD, metabolic associated fatty liver disease; CHG, cholesterol, high–density lipoprotein, and glucose; HC, hip circumference; TG, triglycerides; LDL–C, low–density lipoprotein cholesterol; SBP, systolic blood pressure; DBP, diastolic blood pressure; ALT, alanine aminotransferase; AST, aspartate aminotransferase; GGT, gamma glutamyl transferase; UA, uric acid.

**Table S4** Association between the CHG index and MAFLD after excluding the BMI criterion

| **Exposure** | **OR (95% CI), *P* value** | | |
| --- | --- | --- | --- |
|  | **Model I** | **Model II** | **Model III** |
| CHG (per SD increase) | 3.81 (3.75–3.87) <.001 | 3.24 (3.19–3.29) <.001 | 2.20 (2.16–2.24) <.001 |
| CHG (quartiles) |  |  |  |
| Q1 | 1.0 | 1.0 | 1.0 |
| Q2 | 3.56 (3.41–3.72) | 2.97 (2.84–3.10) | 2.08 (1.99–2.18) |
| Q3 | 10.04 (9.63–10.46) | 7.35 (7.04–7.68) | 3.83 (3.66–4.01) |
| Q4 | 28.41 (27.23–29.64) | 18.84 (18.03–19.69) | 7.31 (6.97–7.67) |
| *P* for trend | <.001 | <.001 | <.001 |

Model I: no covariate adjustment;

Model II: adjusted for sex, age;

Model III: adjusted for sex, age, HC, SBP, DBP, ALT, AST, GGT, UA;

Abbreviations: OR, odds ratio; CI, confidence interval; MAFLD, metabolic associated fatty liver disease; CHG, cholesterol, high–density lipoprotein, and glucose; HC, hip circumference; SBP, systolic blood pressure; DBP, diastolic blood pressure; ALT, alanine aminotransferase; AST, aspartate aminotransferase; GGT, gamma glutamyl transferase; UA, uric acid.

**Table S5** Association between the CHG index with MAFLD after excluding glucose–related criteria

| **Exposure** | **OR (95%CI), *p* value** | | |
| --- | --- | --- | --- |
|  | **Model I** | **Model II** | **Model III** |
| CHG (per SD increase) | 3.81 (3.75–3.87) <.001 | 3.24 (3.19–3.29) <.001 | 2.20 (2.17–2.24) <.001 |
| CHG (quartiles) |  |  |  |
| Q1 | 1.0 | 1.0 | 1.0 |
| Q2 | 3.55 (3.39–3.71) | 2.96 (2.83–3.10) | 2.07 (1.97–2.17) |
| Q3 | 9.99 (9.58–10.42) | 7.32 (7.02–7.65) | 3.80 (3.62–3.98) |
| Q4 | 28.46 (27.27–29.69) | 18.90 (18.08–19.75) | 7.30 (6.96–7.66) |
| *P* for trend | <.001 | <.001 | <.001 |

Model I: no covariate adjustment;

Model II: adjusted for sex, age;

Model III: adjusted for sex, age, HC, SBP, DBP, ALT, AST, GGT, UA;

Abbreviations: OR, odds ratio; CI, confidence interval; MAFLD, metabolic associated fatty liver disease; CHG, cholesterol, high–density lipoprotein, and glucose; HC, hip circumference; SBP, systolic blood pressure; DBP, diastolic blood pressure; ALT, alanine aminotransferase; AST, aspartate aminotransferase; GGT, gamma glutamyl transferase; UA, uric acid.
